# l‐Fucose prevention of renal ischaemia/reperfusion injury in Mice

**DOI:** 10.1096/fj.201901582R

**Published:** 2019-11-27

**Authors:** Mark C. Howard, Christopher L. Nauser, Conrad A. Farrar, Russell Wallis, Steven H. Sacks

**Affiliations:** ^1^ MRC Centre for Transplantation Peter Gorer Department of Immunobiology School of Immunology & Microbial Sciences King's College London London UK; ^2^ Department of Respiratory Science and Infection University of Leicester London UK

**Keywords:** collectin‐11, complement, ischemia/reperfusion, l‐fucose, lectin pathway, transplantation

## Abstract

In a recent study, we identified a fucosylated damage‐associated ligand exposed by ischemia on renal tubule epithelial cells, which after recognition by collectin‐11 (CL‐11 or collectin kidney 1 (CL‐K1)), initiates complement activation and acute kidney injury. We exploited the ability to increase the local tissue concentration of free l‐fucose following systemic administration, in order to block ligand binding by local CL‐11 and prevent complement activation. We achieved a thirty‐five‐fold increase in the intrarenal concentration of l‐fucose following an IP bolus given *before* the ischemia induction procedure ‐ a concentration found to significantly block in vitro binding of CL‐11 on hypoxia‐stressed renal tubule cells. At this l‐fucose dose, complement activation and acute post‐ischemic kidney injury are prevented, with additional protection achieved by a second bolus *after* the induction procedure. CL‐11^−/−^ mice gained no additional protection from l‐fucose administration, indicating that the mechanism of l‐fucose therapy was largely CL‐11‐dependent. The hypothesis is that a high dose of l‐fucose delivered to the kidney obstructs the carbohydrate recognition site on CL‐11 thereby reducing complement‐mediated damage following ischemic insult. Further work will examine the utility in preventing post‐ischemic injury during renal transplantation, where acute kidney injury is known to correlate with poor graft survival.

**Abbreviations**
BSA: bovine serum albumin
CL‐10: collectin‐10
CL‐11: collectin‐11
CRD: carbohydrate recognition domain
DMEM: Dulbecco’s modified eagle medium
EDTA: ethylenediaminetetraacetic acid
ELISA: enzyme‐linked immunosorbent assay
FUT: fucosyltransferase
I/R: ischemia/reperfusion
LAD II: LEUKOCYTE adhesion deficiency II
LMWF: low‐molecular‐weight fucoidan
MASP: mannose binding lectin‐associated serine protease
PSB: phosphate buffered serum
PRM: pattern recognition molecule
PTEC: proximal tubule epithelial cells

## 1 INTRODUCTION

A barrier to successful renal transplantation is the presence of ischemia/reperfusion (I/R) injury, affecting a third of transplants (rising to a half when the donated organ is taken following circulatory arrest).^1^ This in turn results in significant disruption of allograft recovery through delayed graft function and episodes of acute rejection,^2^, ^3^ which increases morbidity and mortality (reviewed in 4). A key effector in I/R injury is the complement system, triggered by recognition molecules that detect ischemic changes and initiate an inflammatory response and downstream tissue damage.^5^

The complement system, an essential part of the innate immune system, is comprised of three activation pathways: classical, lectin and alternative.^6^ It acts through the central complement protein C3 and results in C3b deposition on cellular surfaces. C3b is a marker for membrane attack complex (C5b‐9) formation, which ultimately mediates cell damage.^7^ It also drives an inflammatory response and aids in priming the adaptive immune system for the ultimate removal of antigenic material.^7^, ^8^ The classical and lectin pathways begin with pattern recognition molecules (PRMs) that recognize altered cell surface or foreign ligands. C1q is the principal PRM of the classical pathway,^9^ but there are several PRMs in the lectin pathway (reviewed in 10). The emphasis of our study is on Collectin‐11 (CL‐11), a lectin pathway PRM known to have significant expression throughout major organs,^11^, ^12^ and a major focus of recent work in our lab.^13^, ^14^ CL‐11 is expressed in a number of organs^15^ and within the kidney it is predominantly expressed in the renal tubules but is also present in the glomerular mesangium and epithelium.^16^ Structurally, CL‐11 contains an N‐terminal domain, a collagen‐like region, a neck region, and a carbohydrate recognition domain (CRD),^17^ and is seen in the body as a heterotrimer with collectin‐10 (CL‐10). CL‐10 has also been shown to be found in the kidney at the mRNA and protein level (15, reviewed in 18). Recombinant CL‐11 binds with high affinity to sugar residues such as l‐fucose and d‐mannose but with lower affinity to d‐galactose.^11^ Upon binding, CL‐11, in complex with mannose binding lectin (MBL)‐associated serine protease 2 (MASP‐2), activates complement.^5^, ^19^ Roles for CL‐11 and complement have recently become clear in I/R injury and transplant models.^13^ 3MC (Malpuech‐Michels‐Mingarelli‐Carnevale) syndrome is a rare human disease comprising a variety of developmental abnormalities, such as cleft lip and/or palate, facial dysmorphia, and craniosynostosis, among other features.^20^ Research has shown that 3MC syndrome, although rare, can be caused by deficiency of various lectin pathway components, including CL‐11, MASP‐3 and more recently CL‐10.^20^, ^21^

Work on mouse models of native and transplant kidney I/R injury has shown a key role for the complement system. C3‐deficient mice are protected from I/R injury, demonstrating the central role of C3.^22^ Further research in the same model has both ruled out classical pathway involvement and demonstrated a key role for MASP‐2,^5^ although it should be noted that MASP‐2 can bypass C4 when complexed with MBL.^23^ Most recently, CL‐11 binding has been shown to increase following ischemic injury and is associated with the deposition of complement. This effect is initiated through interaction of CL‐11 with l‐fucose, suggesting that the CRD on CL‐11 recognizes a fucosylated ligand on ischemic tissue.^13^ l‐fucose is a deoxyhexose and a component of a number of glycans that have roles in various biological processes such as host‐microbe interactions, blood transfusion reactions and a number of ontogenic events (24, reviewed in 25). In the kidney, it is detected by plant lectin mainly, if not exclusively, on the proximal tubule. In a murine renal tubule system, removal of fucose by fucosidase blocks the stress‐induced deposition of CL‐11 and, in turn, complement, as does pre‐treatment of recombinant CL‐11 with l‐fucose.^13^ This has led to speculation that I/R injury increases the number of sites to which CL‐11 binds by altering either the presentation or the expression of fucosylated ligand on renal tubular epithelial cell surfaces. It is envisaged that exposed fucosyl residues are recognized and bound by CL‐11. Once CL‐11 binds to these residues it acts to activate complement, in turn resulting in C3 deposition, generation of an inflammatory response and kidney damage. The hypothesis underlying this study is that elevating the level of l‐fucose in the kidneys prior to, and during I/R injury would prevent CL‐11 binding and consequently reduce post‐ischemic injury in a way that could be clinically beneficial. This study uses a well‐established mouse I/R injury to investigate this hypothesis.^5^, ^13^, ^22^

## 2 METHODS

### 2.1 Yeast invertase/collectin‐11 enzyme‐linked immunosorbent assay (ELISA)

MediSorp microtiter plates (Thermo Fisher, Loughborough, UK) were coated overnight at 4°C with 2 µg/mL invertase from *Candida utilis* (Sigma, Gillingham, Dorset, UK) in 0.05 M carbonate‐bicarbonate (Sigma, Gillingham, Dorset, UK). Plates were blocked for 2 hours at room temperature (RT) with tris buffered saline (TBS) /Tw/Ca + 0.1% bovine serum albumin (BSA). The rCL‐11 (3 µg/mL)^26^ and monosaccharide inhibitor (l‐fucose [Sigma, Gillingham, Dorset, UK], d‐mannose [Sigma Gillingham, Dorset, UK], or d‐galactose [Sigma, Gillingham, Dorset, UK]), diluent alone, or 10 mM ethylenediaminetetraacetic acid (EDTA) solution were mixed in 1:1 ratio prior to incubation overnight at 4°C. Further details of the rCL‐11 used throughout this paper are summarised in supplementary Figure 1, which shows it run on a reducing and a non‐reducing gel alongside a gel filtration analysis. It should also be noted that this rCL‐11 is not biotinylated. Plates were incubated with rabbit anti‐human CL‐11 at 1:1000 (Abcam, Cambridge, UK) for 1 hour at RT followed by goat anti‐rabbit‐horseradish peroxidase (HRP) at 1:3000 (Cell Signalling Technologies, London, UK) for 1 hour at RT. The plate was developed with 1‐Step Ultra TMB‐ELISA (Thermo Fisher, Loughborough, UK) and optical density (OD) measured at 450 nm. Concentrations of the buffer components used are as follows: 10 mM Tris, 145 mM NaCl, 0.05% Tween‐20, 2 mM CaCl_2_, pH 7.4.

**Figure 1:**
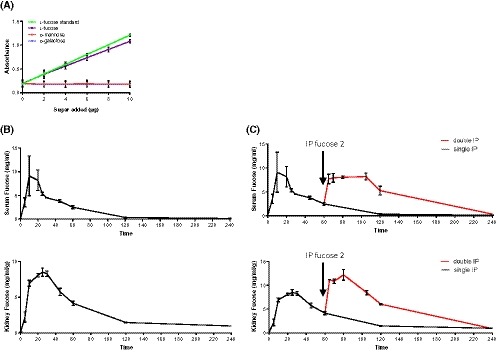
Fucose distribution over time following intraperitoneal (IP) injection. A, Confirmation of K‐fucose kit (megazyme) specificity, showing detection of increasing concentrations of l‐fucose, d‐mannose and d‐galactose compared with manufacturer's l‐fucose standard. B, l‐fucose measurements in serum and kidney following a single IP injection of 100 mg of l‐fucose, at 5, 10, 20, 25 and 30 min and then 15‐min intervals to 60 minutes and 120 and 240 min. C, l‐fucose measurements in serum and kidney samples following a second IP injection of 100 mg l‐fucose (given 60 min after the first dose) at the listed time points following the second dose, up to 60 min**.** Kidneys were homogenized and l‐fucose concentration was determined in homogenized lysate and serum. Kidney values are adjusted to weight of kidney. Each data point is representative of at least two biological repeats and three technical repeats. Error bars on all graphs are standard error of the mean

### 2.2 Animals

CL‐11^−/−^ mice were purchased from Mutant Mouse Resource and Research Centres (MMRRC) (UC Davis, CA, USA)^27^ and backcrossed to C57BL/6 background for four generations. Male mice at 8 weeks of age were used in all experiments with wildtype (WT) offspring of these crosses used as controls.^13^ All experiments adhered to the Animals (Scientific Procedures) Act 1986.

### 2.3 Proximal tubule epithelial cell cultures

Primary Proximal Tubule Epithelial Cell (PTEC) cultures from CL‐11^−/−^ kidneys were prepared as described previously.^28^ Briefly, the cortex was excised from mouse kidney, and digested with 0.1% (w/v) collagenase type II (Worthington Biochemical Corporation, Lakewood, NJ, USA) in Dulbecco's modified eagle medium (DMEM)/F12 and passed through a series of sieves, culminating with a 40‐μm nylon sieve. Cells were cultured on cover slips in 24 well plates preincubated for 2 hours with 1% (w/v) gelatine. Cells were cultured for 5‐7 days in DMEM/F12 medium containing 2% Fetal Calf serum (FCS), 1% Pen/Strep (P/S), insulin (5 μg/mL), transferrin (5 μg/mL), selenium (5 ng/mL), hydrocortisone (40 ng/mL), and triiodothyronine (10^–12^M).

### 2.4 Induction of PTEC stress

PTECs were transferred to a hypoxia chamber (Billups‐Rothenberg, CA, USA) and purged with 5% CO_2_, 1% O_2_ and 94% N_2_ at 20 L/min for 5 minutes, then incubated at 37°C overnight. Cells were allowed to recover at normoxia at 37°C for 2 hours. They were then incubated with 10% serum from either WT or CL‐11^–/–^ mice, or phosphate buffered serum (PBS) as a control. All serums and PBS was diluted in culture medium (without FCS), and the cells were placed at 37°C for 1 hour. Human rCL‐11 (0.9 µg/mL)^26^ was added to CL‐11^‐/‐^ mouse serum 30 minutes prior to addition to PTECs alongside l‐fucose, d‐galactose or culture media (without FCS). Cells were washed, fixed in 4% (w/v) paraformaldehyde (PFA) in PBS for 10 minutes, and immunohistochemistry performed.

### 2.5 Injection of l‐fucose

Mice received intraperitoneal (250 µL; IP) injection of 400 mg/mL l‐fucose or d‐galactose in PBS. Post‐ischemic doses were injected into the abdominal cavity before suturing.

### 2.6 Induction of renal I/R Injury

Injury was induced as previously described.^13^ Mice were anesthetized using isofluorane (Abbott, Maidenhead, UK) and placed on a heating pad. Following laparotomy, renal artery and vein were isolated and occluded using microaneurysm clamps (Codman, UK). Once the clamps were removed, 0.5 mL warm saline, l‐fucose or d‐galactose solution (400 mg/mL) was placed in the abdomen, the incision was sutured, and the animals were allowed to recover in a warm box. Mice were culled 24 hours later. Kidneys and blood were taken for analysis.

### 2.7 Assessment of renal function

Serum was collected from whole blood. A standard kit (BUN Infinity) was used to assess Blood Urea Nitrogen (BUN). All measurements were normalized to the mean WT BUN for a given experiment. Experiments were combined and analyzed as a whole dataset.

### 2.8 Assessment of renal histopathology

Kidneys were fixed in 4% (w/v) PFA in PBS overnight at 4°C then embedded in paraffin. Sections (4‐7 μm) were stained with periodic acid‐Schiff reagent (PAS). Corticomedullary junction tubular injury was graded using a 5‐point scale: 0, normal kidney; 1, <10% necrosis; 2, 10%–25%; 3, 25%–75%; and 4, >75%, in a blinded fashion by two experimenters.^29^

### 2.9 Immunohistochemistry

Kidneys were fixed in Phosphate‐Lysine‐Periodate (PLP) for 2 hours at 4°C and incubated in 13% (w/v) sucrose overnight at 4°C. Kidneys were coronally dissected and embedded in optimal cutting temperature (OCT) compound (CellPath, Newtown, Powys, UK). Frozen sections (4 μm) were air dried overnight at RT, washed and blocked with 20% (v/v) goat or donkey serum in PBS. Sections were incubated with polyclonal rabbit anti‐human C3d (1:200; A0063, Dako, Agilent, Santa Clara, CA, USA), rat anti‐mouse Ly‐6B.2 mAb (1:100; MCA771G, Serotec, Kidlington, UK); rat anti‐mouse CD45 Ab (clone 30‐F11; 1:100; MCD4500, Caltag, Buckingham, UK); or rat anti‐F4/80 Ab (clone CL:A3‐1; 1:100; MCA497, Bio‐rad, Hertfordshire, UK) followed by FITC‐conjugated goat anti‐rabbit IgG Ab (1:100; 111‐095‐144, Stratech Scientific, Cambridge, UK), goat anti‐rat Alexa Fluor 488 (1;200; a11006, Invitrogen, Thermo Fischer, Loughborough, UK) or donkey anti‐rabbit Alexa Fluor 594 (1:200; 406418, Life Technologies, Thermo Fischer, Loughborough, UK) at RT for an hour. Antibodies were diluted in 20% blocking buffer and nuclei stained with 4′,6‐diamidino‐2‐phenylindole (DAPI) (1:10 000; Life Technologies, Thermo Fischer, Loughborough, UK).

### 2.10 l‐Fucose analysis

Serum was prepared as described above. Whole kidney was homogenized in 1M perchloric acid. Homogenate was adjusted to pH 8.0, volume adjusted to equalize samples and incubated on ice. Next, 1 mL of homogenate was spun for 10 minutes and the supernatant applied to a K‐fucose kit (Megazyme, Wicklow, Ireland). Samples were added to a solution containing nicotinamide adenine dinucleotide phosphate (NADP^+^) in a 96 well plate and preincubated at 37°C for 3 minutes. l‐fucose dehydrogenase was then added and the plate was incubated for 15 minutes at 37°C. The product of this reaction was then measured by absorbance at 340 nm and l‐fucose concentrations calculated from a standard curve made using known l‐fucose quantities. Serum was adjusted to mg/mL and kidney fucose was normalized to kidney weight ([mg/mL]/g).

### 2.11 Imaging infiltrating cells and C3d expression

Tissue staining was visualized using microscopy and quantified using ImageJ (NIH). Infiltrating leukocytes within the corticomedullary junction were counted at a magnification of 20×, and the percentage of C3d deposition per image was calculated by setting a threshold for background removal. From a minimum of three fields per animal, the number of immune cells/C3d deposition was determined.

### 2.12 Statistics

Data are shown as the mean ± SEM or as values for individual data points. For comparisons between two groups, an unpaired, 2‐tailed Student's t test was used, and a *P* value of less than .05 was considered significant. For three or more groups, pairwise comparisons of specific groups were undertaken and again a *P* value of less than .05 was considered significant. All statistics were performed using GraphPad Prism, version 8 (GraphPad Software).

## 3 RESULTS

### 3.1 Levels of administered l‐fucose rapidly rise and fall in serum but remain stable in the kidney over several hours

We applied increasing concentrations of l‐fucose alongside two alternative sugars to a commercial fucose kit to demonstrate its specificity (Figure 1A). We then took readings of serum and kidney l‐fucose levels at intervals following IP injection (Figure 1B). l‐fucose rapidly distributes to both the serum and the kidney and then in the case of serum levels return to pre‐IP injection levels at 2 hours. However, in the kidney l‐fucose levels remain at a low, but above baseline level past 4 hours. At early time‐points the variability of the serum L‐fucose measurements was considerable, especially between 5 and 20 minutes. Biological repeats were more consistent at subsequent time‐points. The initial inter‐mouse variability could have been due to technical differences, such as variations in pressure of injection and exit from the peritoneal cavity into the blood, which evened out over time with homeostatic control.

Serum l‐fucose levels peaked at 10 minutes at 9.1 (±4.2) mg/mL before rapidly decreasing to 4.5 (±0.01) mg/mL at 30 minutes and 2.5 (±0.29) mg/mL at 60 minutes. The kidney concentration took longer to reach its maximal l‐fucose concentration of 8.35 (±0.26) (mg/mL)/g at 25 minutes. But the kidney sustained these levels, with the intrarenal l‐fucose concentration being 8.30 (±0.59) (mg/mL)/g at 30 minutes and 4.15 (±0.31) (mg/mL)/g after 60 minutes. Thus, the kidney concentration of l‐fucose following 400 mg/mL IP administration was maintained at a level of at least 46 times (peaking at over 95 times) the normal kidney concentration (0.090 [±0.016] [mg/mL]/g) for over 1 hour and remained at 10 times the basal level after 4 hours, when the serum concentration had returned to normal (0.070 [±0.010] mg/mL). A second injection 60 minutes after the first maintained the raised concentration of l‐fucose in the kidney for a longer period, while similarly transient kinetics occurred in the serum (Figure 1C). The peak after the second injection is not only substantially higher (12.2 [±1.1] [mg/mL]/g) than that of the single injection, but the duration of supraphysiologic fucose levels is extended beyond 120 minutes, where the l‐fucose remains at 6.04 (±0.14) (mg/mL)/g. It should be noted that the peak intrarenal level of l‐fucose (between 2 and 2.6 mg/mL) occurs between 15 and 30 minutes (and between 70 and 105 minutes after a second dose); a working concentration between 12 and 16 mM. Additionally, after two l‐fucose injections, the mouse kidney remained at a level above 8 mM until 120 minutes post‐first injection.

### 3.2 l‐fucose blocks CL‐11 binding to a similar extent as d‐mannose but a greater extent than d‐galactose on immobilised yeast invertase

**Figure 2:**
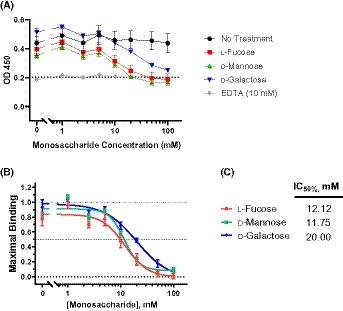
Monosaccharide Inhibition of CL‐11 binding. A, Level of CL‐11 binding to immobilized yeast invertase measured using enzyme‐linked immunosorbent assay (ELISA). l‐fucose, d‐mannose and d‐galactose were added at increasing values to determine blocking of CL‐11. Values are blank‐corrected OD_450_ measurements. Dotted line is the average of all blank‐corrected EDTA‐treated wells which corresponds to a functionally zero value for CL‐11 binding (which is known to be calcium‐dependent). B, Sugar inhibition of CL‐11 binding to yeast invertase. Lower dotted line represents 100% inhibition, middle dotted line represents 50% inhibition, and higher dotted line represents no inhibition. C, Table of half maximal inhibitory concentration (IC_50_) values calculated form data presented in (A) and (B). Each data point is representative of at least two biological repeats and three technical repeats. Error bars on all graphs are standard error of the mean. IC_50_ was calculated using a four‐parameter logistic curve, while graphs show a best fit line

To establish a functional concentration of l‐fucose needed to block CL‐11 binding, we performed in vitro sugar blocking studies. Utilizing a previously published ELISA method^26^ of CL‐11 binding to a known ligand, we added a number of sugars to establish the extent of CL‐11 blockade (l‐fucose, d‐Mannose and d‐galactose) up to the level of a no Ca^2+^(EDTA) control (Figure 2). The half maximal inhibitory concentration (IC_50_) for l‐fucose and d‐galactose was 12.12 mM and 11.75 mM respectively, while d‐galactose was approximately half as effective (IC_50_ of 20). This agrees with known affinities of CL‐11 for different sugars^11^ and shows that d‐galactose is a suitable control molecule for all other experiments in this study. The IC_50_ of l‐fucose in our ELISA (12.12 mM) approximates the level measured in the kidney following IP injection (and indeed for the double IP injection). Importantly, as clamping the renal blood vessels at 60 minutes after the IP injection is assumed to block the exit of fucose from the kidney, the levels of l‐fucose are likely to remain between 8 mM and 16 mM during the first hour of reperfusion.

### 3.3 In a cell culture system, l‐fucose blocks rCL‐11‐induced deposition of complement

**Figure 3:**
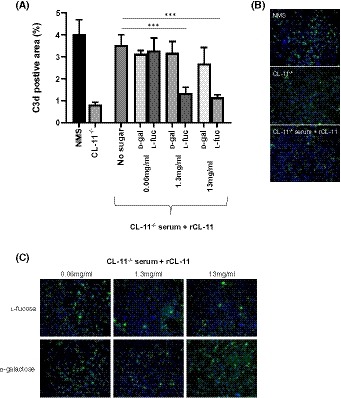
Quantification of C3d deposition on hypoxia‐stressed mouse PTECs. CL‐11^−/−^ mouse PTECs were stressed by 16 hours of hypoxia and this was followed by addition of 10% normal mouse serum (NMS) or CL‐11^−/−^ serum. To CL‐11^−/−^ serum was added 0.9 µg/mL rCL‐11 and different levels of l‐fucose or d‐galactose, or no sugar. A, Percentage of C3d‐positive staining determined from 12 individual fields and representative of three independent experiments. B, and C, representative images of cells used for quantification in (A). Each data point is representative of at least three biological repeats and three technical repeats. Error bars on all graphs are standard error of the mean. ***P < .005

PTECs isolated from CL‐11^−/−^ mice were exposed to hypoxic stress. The subsequent addition of WT serum (ie, containing CL‐11) to the culture led to cell‐surface deposition of C3d, consistent with complement activation, while CL‐11^−/−^ serum does not, as previously shown.^13^ Adding 0.9 µg/mL of rCL‐11 to CL‐11^−/−^ serum, ensured the presence of rCL‐11 alongside other complement factors, and this resulted in C3d deposition. It should be noted that we use human rCL‐11 on these cells as we have previously found this to be effective in activating complement.^13^ It has been shown in the literature that human and mouse CL‐11 have the same preference for l‐fucose over other sugar residues,^11^, ^12^ and consequently we are confident that these findings using human rCL‐11 are applicable to the role of CL‐11 in the mouse kidney. To this was added l‐fucose or d‐galactose control in various amounts to give a final concentration of 0.06 mg/mL (0.37 mM), 1.3 mg/mL (8 mM) and 13 mg/mL (80 mM), where 0.06 mg/mL (0.37 mM) corresponds to the normal serum level, 1.3 mg/mL (8 mM) to the measured level sustained in the kidney for 60 minutes following two IP injections (Figure 1) and 13 mg/mL (80 mM) to 10 times this amount. The presence of l‐fucose in normal serum levels did not reduce C3d deposition, but at supraphysiologic levels of l‐fucose (1.3 mg/mL), C3d deposition was reduced by approximately 60%, compared to d‐galactose ‐treated or no‐added‐sugar controls (Figure 3). A similar level of complement inhibition occurred with the highest dose of l‐fucose (13 mg/mL), suggesting that a local tissue concentration of 1.3 mg/mL would be a reasonable therapeutic target to block CL‐11‐associated complement deposition.

### 3.4 Supraphysiologic levels of renal l‐fucose demonstrate a protective effect against I/R injury

**Figure 4:**
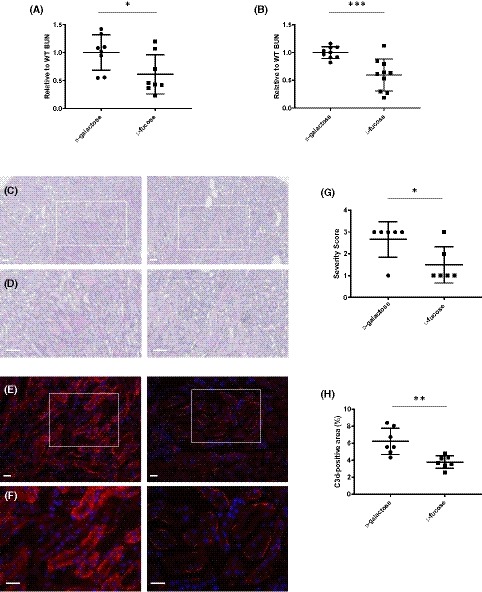
l‐Fucose has a protective effect on mouse kidneys after IR injury induction. Individual mice were dosed intraperitoneally with 100 mg of l‐fucose or d‐galactose 1 h before induction of 30 min bilateral renal ischemia (A) or both 1 hour before clamping and immediately after removal of clamps (B). Renal function (BUN) was measured in serum 24 hours post‐reperfusion and normalized within each individual experiment before being combined. Mean BUNs were (A) 33.68 mmol/L and 20.21 mmol/L, and (B) 30.98 mmol/L and 19.68 mmol/L for d‐galactose and l‐fucose, respectively. C and D, representative images of PAS staining of mouse kidneys from (B) (n = 6 mice/group), at 10x (C) or 20x (D) magnification. E and F, representative images of C3d staining (red) of mouse kidneys from (B) (n = 7 mice/group), at 10× (E) or 20× (F) magnification. G, Quantification of C3d staining shown in E. Scale bars = 100 µm. Error bars on all graphs are standard error of the mean. *P < .05, **P < .01, ***P < .005

To investigate the potential therapeutic application of l‐fucose in renal I/R injury we used an established murine model. This model is characterized by an increased expression of fucosylated molecules alongside CL‐11 bound to the basolateral surface of the proximal renal tubules at 24 hours of reperfusion following 30 minute ischemia.^13^ Furthermore, the model elicits a role for local complement in the pathogenesis of the ischemic injury^29^ and a role for CL‐11.^13^ We tested two regimens of l‐fucose administration. First, mice received a single IP bolus of l‐fucose 60 minutes prior to induction of ischemia. The resulting concentration of l‐fucose during the 30 minutes of ischemia was 0.62 (±0.014) mg/mL (or 4.15 (±0.31) (mg/mL)/g normalized to kidney weight) or a working concentration of 3.84 mM, which gradually fell after reperfusion (Figure 1B). Under these conditions, there was a significant reduction in the mean kidney injury compared with d‐galactose‐injected controls, despite notable inter‐mouse variation (mean BUNs of 33.68 mmol/L and 20.21 mmol/L for d‐galactose and l‐fucose, respectively; Figure 4A). In the second regimen, mice received two successive IP doses of l‐fucose, 60 minutes prior to I/R injury as before, and a second dose immediately following reperfusion. As a result, the renal l‐fucose concentration was at least 10 mM by 10 minutes after reperfusion and remained above 11 mM from 20 minutes, therefore achieving levels above those of the previous experiment over a longer period. Analysis of kidney function (Figure 4B) (mean BUNs of 30.98 mmol/L and 19.68 mmol/L for d‐galactose and l‐fucose respectively) and tubule necrosis (Figure 4C,D) showed a reduction of acute kidney injury in l‐fucose ‐treated mice. A decrease in complement (C3d) deposition (Figure 4E,F) in l‐fucose ‐treated mice showed a parallel reduction in complement activation on the vulnerable segments of renal tubules, as previously described.^13^, ^22^, ^29^

### 3.5 Pre‐treatment with l‐fucose reduces immune cell infiltration associated with complement system activation

**Figure 5:**
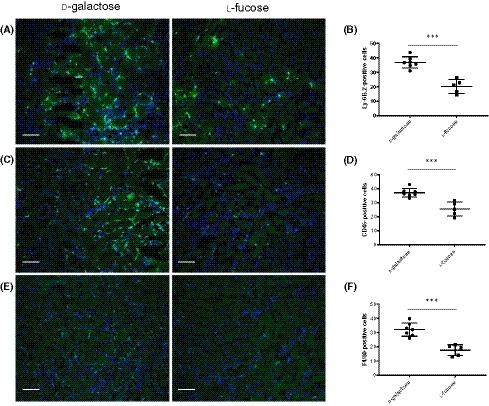
Impact of l‐fucose on immune cell infiltration after 30 min of ischemia. Individual mice were dosed intraperitoneally with 100 mg of l‐fucose or d‐galactose 1 h before induction of 30 min bilateral renal ischemia and again at the time the clamps were removed. Renal function (BUN) was measured in serum 24 h post‐reperfusion. A, Representative images of immunochemical staining of neutrophils in kidneys sections from mice with l‐fucose or d‐galactose IP injections. B, Quantification of neutrophils in kidney sections from the mice represented in A. C, Representative images of immunochemical staining of leukocytes in kidney sections from mice with l‐fucose or d‐galactose IP injections. D, Quantification of leukocytes in kidney sections from the mice represented in C. E, Representative images of immunochemical staining of macrophages in kidneys sections from mice with l‐fucose or d‐galactose IP injections. F, Quantification of macrophages in kidney sections from the mice represented in E. Scale bars = 100 µm. All images are representative of staining (n = 7 and n = 5 mice/group for d‐galactose and l‐fucose groups respectively). Error bars on all graphs are standard error of the mean. ***P < .005

To further corroborate the effect of l‐fucose pre‐treatment on complement‐mediated inflammation, we analyzed the tissue infiltrate of immune cells. Using markers for neutrophils (Ly‐6B.2), leukocytes (CD45) and macrophages (F4/80) we showed a significant reduction in the number of immune cells infiltrating into the corticomedullary junction (Figure 5). Thus, in addition to blocking activation of the complement, l‐fucose blockade of CL‐11 also reduces the recruitment of immune and inflammatory cells.

### 3.6 The protective effect of l‐fucose is dependent on CL‐11 and the complement system

**Figure 6:**
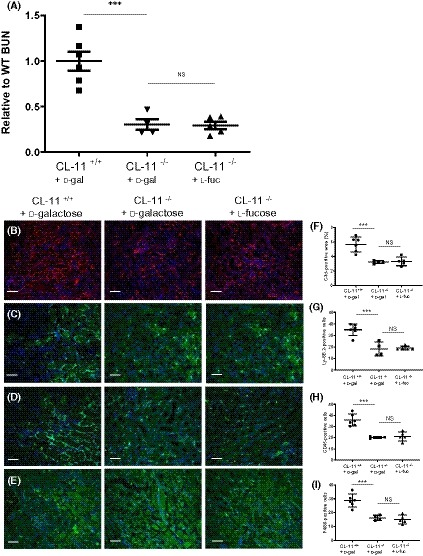
Absence of CL‐11 renders l‐fucose therapy ineffective in renal I/R injury. Individual mice were dosed intraperitoneally with 100 mg of l‐fucose or d‐galactose 1 h before induction of 30 min bilateral renal ischemia and again at the time the clamps were removed. Renal function (BUN) was measured in serum 24 h post‐reperfusion (A). Mean BUNs were 45.77 mmol/L, 13.98 mmol/L and 13.56 mmol/L for WT with d‐galactose, CL‐11^−/−^ with d‐galactose and CL‐11^−/−^ with l‐fucose, respectively. B, Representative images of fluorescent C3 staining of mice in A. F, Quantification of C3d staining in B. C, Representative images of immunochemical staining of neutrophils in kidneys sections from the mice in A. G, Quantification of neutrophils in kidney sections from the mice represented in A and C. D, Representative images of immunochemical staining of leukocytes in kidneys sections from the mice in A. H, Quantification of leukocytes in kidney sections from the mice represented in A and D. E, Representative images of immunochemical staining of macrophages in kidneys sections from the mice in A. I, Quantification of macrophages in kidney sections from the mice represented in A and E. Scale bars = 100 µm. All images are representative of staining (n = 6 for CL‐11^+/+^ d‐galactose, n = 4 for CL‐11^−/−^ D‐galactose, and n = 5 for CL‐11^−/−^ l‐fucose). Error bars on all graphs are standard error of the mean. *P < .05, ***P < .005

According to our hypothesis, fucose treatment acts by obstructing CL‐11 binding to target ligand on ischemic renal tissue, and therefore the administration of l‐fucose should be ineffective when CL‐11 is absent. In contrast, if fucose acted through a CL‐11‐independent mechanism, an additive effect would be evident. The treatment of CL‐11‐deficient mice with l‐fucose afforded no additional protection against the development of renal I/R injury, compared with d‐galactose ‐treated mice (Figure 6), including loss of renal function (mean BUNs were 45.77 mmol/L, 13.98 mmol/L and 13.56 mmol/L for WT d‐galactose, CL‐11^−/−^ d‐galactose and CL‐11^−/−^ l‐fucose respectively [Figure 6A]), renal complement (C3d) deposition (Figure 6B) and leukocyte infiltration (Figure 6C–E). Not only does this suggest that the effect of l‐fucose treatment was CL‐11‐dependent, but it also indicates that there is no major independent effect of the treatment on glycan‐receptor interactions that mediate leukocyte migration (reviewed in 30). These findings are consistent with a pharmacological effect of l‐fucose primarily on a CL‐11‐mediated pathway of post‐ischemic renal injury in our model.

## 4 DISCUSSION

Our study provides a rational basis for considering l‐fucose a potential therapeutic approach to the inhibition of renal I/R injury. Previous work has shown that CL‐11 plays a key role in the initiation of complement‐mediated damage to renal tubules following I/R injury and this is likely due to the binding of CL‐11 to a fucosylated ligand. In vitro analysis previously showed that free l‐fucose inhibited the binding of recombinant CL‐11 to a proposed damage‐associated ligand.^13^ Therefore, would saturating the kidney with an excess of free l‐fucose, prior to I/R injury, block CL‐11 binding to its damage‐associated ligand and likewise diminish complement‐mediated injury? Our data establishes that an excess of free fucose in the kidney following systemic administration is sustainable for a critical period and protects against development of hypoxia‐induced acute kidney injury.

Throughout this discussion we use both the words inhibit and block. We specifically use block as we refer to the physical obstruction of the CRD on CL‐11 by l‐fucose but this should not be understood to mean a complete blockade of all CL‐11 functions in these circumstances. The extent of the block varies depending on levels of l‐fucose as the discussed below.

The concentration of soluble l‐fucose in the renal cortex following a double IP bolus injection (Figure 1) is sufficient for in vitro blockade of CL‐11 binding on immobilized ligand (Figure 2) and inhibition of complement activation on hypoxic renal tubule cells (Figure 3). When l‐fucose was added at a normal serum level (0.06 mg/mL), complement deposition was not blocked; however when added at 1.3 mg/mL (8mM), corresponding to the kidney concentration at 60 minutes following double IP injection (Figure 1), complement deposition was mostly prevented (Figure 3A). Higher l‐fucose concentrations did not substantially increase the blocking effect. In contrast, a monosaccharide such as d‐galactose with only weak binding to the CRD on CL‐11 had no major inhibitory effect.

In our ELISA blocking assay (Figure 2) we use a rCL‐11 that has been used both in our lab^13^ and several others.^26^, ^31^ It is a recombinant homo‐oligomer generated by expression of the CL‐11 cDNA (isoform a). It consists primarily of monomers and dimers (and smaller amounts of larger oligomers) of a subunit composed of three polypeptide chains (Supplementary Figure 1). Previous studies have shown that it binds to MASPs with high affinities,^31^ similar to those of MBL‐MASP and ficolin‐MASP complexes.^32^, ^33^ Moreover, rCL‐11 activates complement with comparable activity to recombinant human MBL (~2.5‐fold lower), in human serum depleted of endogenous lectins.^26^ This ELISA establishes l‐fucose blocks rCL‐11 (Figure 2) at a concentration that approximates the amount of fucose present in the kidney at the induction of I/R injury. Mannose blocks rCL‐11 binding to a similar extent as l‐fucose (IC_50_ of 11.75 and 12.12, respectively), approximating that previously published.^26^ Meanwhile d‐galactose is about half as effective, justifying the use of d‐galactose as a control throughout this study. It has been reported that d‐mannose and l‐fucose blocking of CL‐11 binding are as effective as each other, while d‐galactose is considerably less effective in blocking CL‐11 binding to mannose‐BSA.^11^

Thus, in vitro, l‐fucose blocks the binding of CL‐11 to immobilized ligand and inhibits CL‐11‐mediated complement deposition on cultured cells, consistent with the protective effect of systemic administration of l‐fucose we found in vivo. These data support the proposed therapeutic mechanism in which the local tissue concentration of l‐fucose is elevated to a level that competitively blocks CL‐11 binding to ischemic tissue, eliminating the trigger for complement activation. There is a possibility that the mode of l‐fucose action is through a different pathway than via CL‐11 signalling and consequently we looked at its effect in CL‐11^−/−^ mice. We observed no additional protective effect in CL‐11‐deficient mice treated with l‐fucose; specifically complement activation and leukocyte infiltration were no less in the l‐fucose ‐treated CL‐11‐deficient mice than in d‐galactose‐treated controls (Figure 6). The data are consistent with a decoy mechanism whereby the raised concentration of free l‐fucose in the renal interstitium blocks CL‐11 binding to fucosylated ligand and so prevents triggering of complement activation by the lectin pathway. We did consider using fucosyltransferase (FUT)‐deficient mice as a means to test the impact of fucosylation on ischaemic injury; however, the wide range of molecules and biological functions likely to be affected by deficient fucosylation even with a specific FUT deletion would be unlikely to localize the effect of ligand disruption to collectin‐11. Rather, the findings in CL‐11 deficient mice treated with l‐fucose are against a major effect of the sugar on leukocyte‐receptor‐ligand interaction mediating leukocyte migration and are in keeping with a primary mode of action of l‐fucose blocking the carbohydrate recognition domain of CL‐11. Further work to make this definitive will require increased knowledge of the kidney glycome as well as details of the specific ligand for CL‐11 in this process.

In achieving sustainable local tissue concentrations of l‐fucose following IP administration, we noted important differences between the two regimens using single (Figure 4A) and repeated (Figure 4B) doses. Longer exposure time to maintain an effective inhibitory concentration occurred with repeated dosing of l‐fucose compared to a single dose. The data indicate that with a single IP injection of l‐fucose, CL‐11 binding is significantly but not consistently blocked (Figure 4A) (*P* = .0351). If it is assumed that l‐fucose levels remain broadly constant during ischemia, since both renal artery and vein are occluded, then upon the removal of renal clamps and the commencement of reperfusion, the second dose causes a substantial increase in kidney l‐fucose as well as a prolonged exposure to this higher concentration, which returns gradually to steady state over the next 24 hours (data not shown). This increased kidney l‐fucose causes a more significant reduction in kidney damage as well as a reduction of inter‐mouse variation (Figure 4B) (*P* = .001). The finding of normal tissue concentration of l‐fucose in d‐galactose‐treated mice confirmed that the elevation in l‐fucose was related to l‐fucose injection, rather than ischemic damage *per se*.

The results presented here have obvious clinical implications for the prevention of I/R injury and delayed graft function.^34^ Delayed renal graft function is a common manifestation of acute post‐ischemic kidney injury, especially in diseased‐donor organ transplantation, and has adverse effects on graft immunogenicity and long‐term graft survival.^35^ The potential value for therapy is enhanced by lack of toxicity observed in our mice and in previously reported animal and human studies. l‐fucose is a natural constituent of many glycans that are presented at the cell membrane of mammalian cells.^25^ Previous cancer studies have shown that IP injections of l‐fucose are not detrimental in mice up to a level of 5 g/kg (approximately 160 mg).^36^ Additionally, low‐molecular‐weight fucoidan (LMWF), a group of fucose‐enriched sulphated polysaccharides extracted from seaweed, was shown to have a protective effect on I/R injury in mice,^37^ though the mechanism was not fully explained. The circulating levels of l‐fucose following IP administration in our studies were between 40 and 130 times the normal level in mice, and these equate to the levels in human subjects treated with l‐fucose in previous studies.^38^ One hour following oral fucose administration levels were between 20× and 40× normal serum levels with a maximum of 75× (for leukocyte adhesion deficiency II (LAD II)^38^, ^39^), approximating our findings detailed here. In particular, no change in animal behavior or feeding habit was evident in our mice.

Further optimization of the fucose‐delivery protocol may be possible, depending on the application being considered. For example, a constant infusion protocol could lead to steady‐state tissue‐fucose concentration for a longer period and possibly reduce inter‐subject variation and increase the degree of protection. Pre‐emptive treatment of donated kidneys that are intended for use in organ transplantation may also be feasible.

Our results validate the general concept that fucosylated structures mediate the binding of CL‐11 to hypoxic renal tubule cells and that soluble l‐fucose is a competitive inhibitor that binds to the carbohydrate recognition domain of CL‐11. The results showing a therapeutic effect of supraphysiological levels of l‐fucose within the renal cortex, combined with the in vitro effect on the cell binding properties of CL‐11, make a strong case for further investigation. This is particularly relevant in renal transplant ischemia reperfusion injury, where the risk of delayed graft function is ever present given the more frequent use of marginal donors.

## AUTHOR CONTRIBUTIONS

M. Howard, C. Nauser, C. Farrar, R.Wallis, and S. Sacks conceived and planned the experiments; M. Howard, C. Nauser, R.Wallis, and C. Farrar carried out the experiments; M. Howard and C. Nauser carried out statistical analysis; M. Howard, C. Nauser, C. Farrar, R.Wallis, and S. Sacks contributed to the interpretation of the results; M. Howard led the writing of the manuscript. All authors provided critical feedback and helped shape the research, analysis, and manuscript.

## DISCLOSURES

None.
