# Supplementary material for: l‐Fucose prevention of renal ischaemia/reperfusion injury in Mice
Source: FASEB J. 2019 Nov 27;34(1):822–34. doi: 10.1096/fj.201901582R (PMC6972607; doi:10.1096/fj.201901582R)
Supplement: Supplementary file 1 [file FSB2-34-822-s001.pptx]

## Slide 1
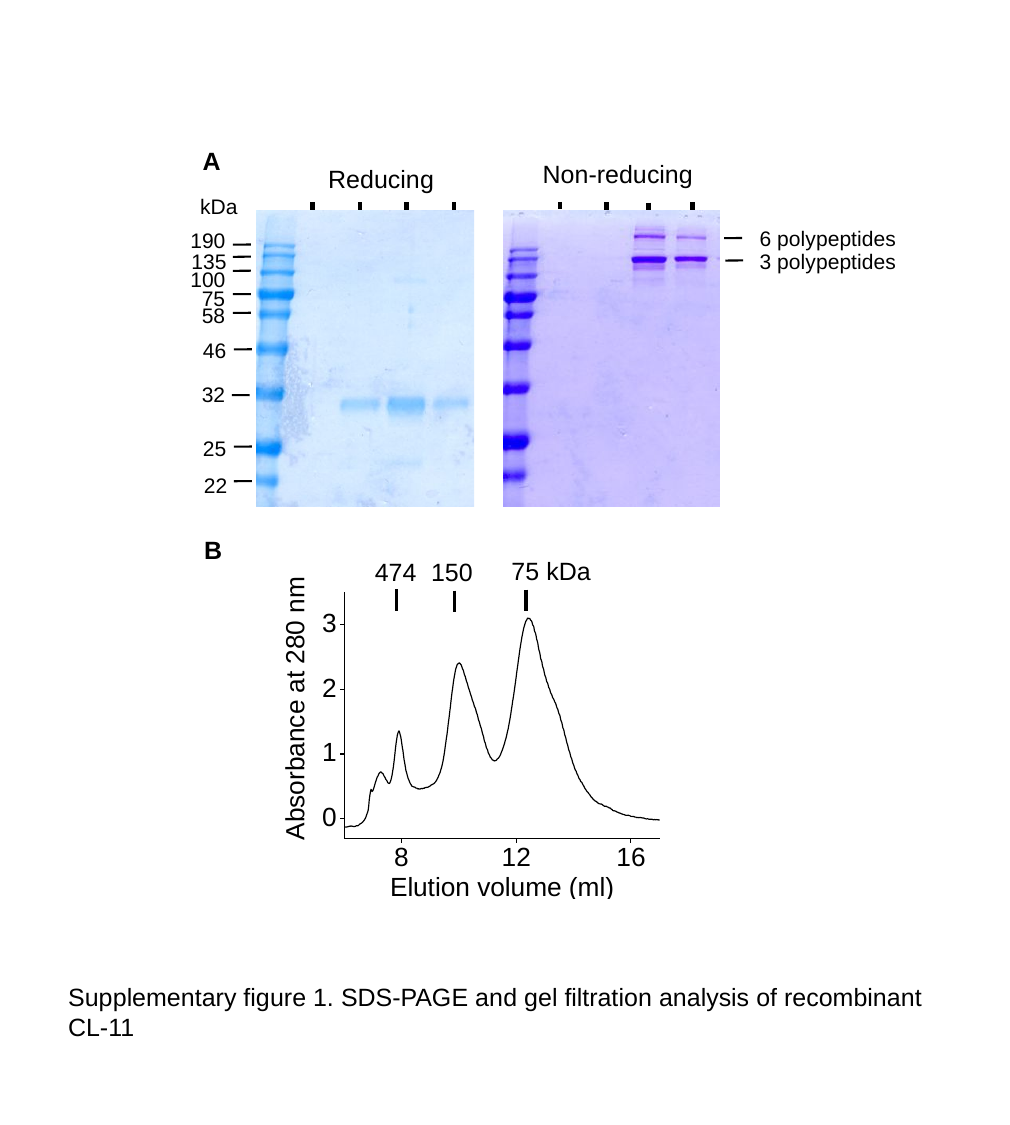

A
Non-reducing
Reducing
kDa
6 polypeptides
190
135
3 polypeptides
100
75
58
46
32
25
22
B
75 kDa
474
150
Supplementary figure 1. SDS-PAGE and gel filtration analysis of recombinant CL-11
