# Supplementary material for: l‐Fucose prevention of renal ischaemia/reperfusion injury in Mice
Source: FASEB J. 2019 Nov 27;34(1):822–34. doi: 10.1096/fj.201901582R (PMC6972607; doi:10.1096/fj.201901582R)
Supplement: Supplementary file 2 [file FSB2-34-822-s002.docx]

**Supplementary figure 1.** SDS-PAGE and gel filtration analysis of recombinant Collectin-11 (rCL-11). **A)** representative SDS-PAGE of purified rCL-11 on 12% gels under reducing and non-reducing conditions. Each lane is a consecutive elution fraction purified from a mannose-sepharose column (2 separate preparations). CL-11 consists of disulphide-linked oligomers of the CL-11 polypeptide (~32 kDa). The predominant species are 3 and 6 disulphide-linked polypeptides, with lower amounts of larger oligomers. **B)** Gel filtration of rCL-11 on a Superdex 200 column (10/300) in 50 mM Tris pH 7.5 containing 150 mM NaCl and 1 mM EDTA. Absorbance values are in milliabsorbance units with a pathlength of 0.5 cm. rCL-11 elutes as multiple species that probably comprise monomers (12.5 mL), dimers (10.5 mL) and larger oligomers (7.5 - 8 mL) of the trimeric subunit.
